# Effects of Scallop Visceral Mass and Mantle as Dietary Supplements on the Growth, Immune Response and Intestinal Microflora of Juvenile Sea Cucumber *Apostichopus japonicus*

**DOI:** 10.3390/biology12091239

**Published:** 2023-09-14

**Authors:** Yu Yu, Mengshu Wang, Yichao Ren, Xin Wang, Xiangyun Ge, Kecheng Li

**Affiliations:** 1 School of Marine Science and Engineering, Qingdao Agricultural University, Qingdao 266109, China; yuyu@qau.edu.cn (Y.Y.); wangmengshu0725@163.com (M.W.); wangx8253@163.com (X.W.); 17346872166@189.cn (X.G.); 2 CAS and Shandong Province Key Laboratory of Experimental Marine Biology, Institute of Oceanology, Chinese Academy of Sciences, Qingdao 266071, China; 3 Laboratory for Marine Drugs and Biological Products, Qingdao National Laboratory for Marine Science and Technology, Qingdao 266237, China

**Keywords:** scallop visceral mass, scallop mantle, supplementation, fatty acid, immune gene, intestinal microbe

## Abstract

**Simple Summary:**

During scallop production, huge amounts of scallop visceral mass and mantle are treated as waste and discarded every year. However, scallop visceral mass and mantle have high contents of protein and other nutritional substances. Hence, this study aimed to add them into the feed of juvenile sea cucumber (*A. japonicus*), culture for 40 days, and then study the effects on the growth and health of the sea cucumber. The results showed that the addition of scallop visceral mass significantly improved the growth rate of juvenile *A. japonicus* within 20 days, and also markedly increased the contents of ω-3 fatty acids including the EPA and DHA, which is an indication of good health condition and high commercial value of sea cucumber. Furthermore, the addition of scallop visceral mass and mantle enhanced the immune defense in the sea cucumber, increased the microbial diversity and the abundance of beneficial microbes including *Bifidobacteriaceae*, *Streptomycetaceae*, *Clostridiaceae* and *Rhizobiales* in the gut of the sea cucumber.

**Abstract:**

Scallop visceral mass and mantle are aquatic byproducts and waste, but they have high contents of protein. In this study, scallop visceral mass and mantle were used as supplements in the diet of juvenile sea cucumber (*A. japonicus*) and their effects on the growth, fatty acid and amino acid compositions, the non-specific immune responses and the intestinal microflora of *A. japonicus* were investigated through a 40 d feeding experiment. The results showed that dietary supplementation of scallop visceral mass significantly accelerated the specific growth rate (SGR) of juvenile *A. japonicus* by 3 times within 20 days, and also raised the contents of ω-3 fatty acids including EPA and DHA and the ω-3/ω-6 ratio of the sea cucumber tissue, which is favorable to the health and commercial value of the sea cucumber. Furthermore, it was found that the supplementation of scallop visceral mass and mantle stimulated the expression of immune-related genes and enhanced the immune defense in *A. japonicus*. Scallop visceral mass and mantle supplementation also increased the microbial diversity and the abundance of beneficial microbes including *Bifidobacteriaceae*, *Streptomycetaceae*, *Clostridiaceae* and *Rhizobiales* in the gut of *A. japonicus*. This study reveals the beneficial effects of dietary supplementation of scallop visceral mass and mantle on the growth of juvenile *A. japonicus*, which might be a promising way to reutilize this scallop waste and raise its economic value.

## 1. Introduction

The annual aquaculture production of scallops is increasing and reaches nearly 2 million tons in China. Scallop visceral mass and mantle are the main byproducts during scallop processing which are considered low value and usually discarded as waste [1]. However, the scallop visceral mass and mantle have a high content of protein and contain plenty of bioactive substances with anti-virus, anti-tumor and immunostimulatory activities [2,3]. It was reported that scallop muscle contained tropomyosin and actin [4], and functional products were extracted from the scallop mantle, such as the protein hydrolysate exhibiting abilities to reduce hydroxyl and DPPH radicals. The reuse of scallop visceral mass and mantle has attracted wide attention, which is also needed to lessen the burden on the environment.

The sea cucumber *Apostichopus japonicus* is one of the most commercially important species in Asia countries especially in China due to its nutritional value and therapeutic properties [5]. The annual production was 196,564 tons with a value of more than 50 billion US dollars in 2021 [6]. However, with the development of aquaculture, disease outbreaks that result in high mortality have become a bottleneck in the sustainable development of the aquaculture industry. Antibiotics such as furans quinolones and oxytetracyclines are commonly used for the prevention and control of bacterial infections in sea cucumber cultures [7]. Farmers have realized that disease prevention is more important than cure. Therefore, immunostimulants have been used to enhance the immune responses of *A. japonicus*. Dietary intervention is an environment-friendly and effective way of improving the health of sea cucumbers from both the nutritional and immunological perspectives. 

*Chlamys farreri* is a high-economic shellfish species commonly cultured on the East Asian coast, and full utilization of *C. farreri* has attracted increasing attention. Scallop mantle has a high content of protein and might be used as a protein source in the diet of *A. japonicus* [8]. And scallop mantle subjected to enzymolysis could also modulate the nonspecific immune responses of *A. japonicus* [8]. Scallop visceral mass was reported to be rich in unsaturated fatty acids [9], but it has not been used in the feed of sea cucumbers. Therefore, it is necessary to comprehensively investigate the effects of scallop mantle and visceral mass on the growth, immunology and intestinal microbiota of *A. japonicus*, which is important for the sustainable utilization of scallop bio-resources, as well as a guidance for the aquaculture and feeding of sea cucumber.

## 2. Materials and Methods

### 2.1. Diet Preparation

Fresh Scallop *C. farreri* (size: 5.7–6.4 cm; weight: 16–33 g) was purchased from a local market in March in Qingdao City, China. The visceral mass and mantle of the *C. farreri* were separated, homogenized and then dried at 45 °C. Then, they were ground into fine powder for diet preparation. A powder composed of *Sargassum thunbergii* and sea mud (4:6) was used as the basal diet. A previous report stated that 2% was the optimal supplementary concentration of enzymatically hydrolyzed scallop visceral protein powder for broiler chicken feed [10]. Through a pre-experiment for one week, we also observed that the sea cucumbers had better feeding and excretion status with the supplementation of 3.5% of scallop visceral mass and scallop mantle powder in their diet. Therefore, three feeding groups were designed: (1) CK with the basic diet; (2) SV with the basal diet adding 3.5% of scallop visceral mass powder; and (3) SM with the basal diet adding 3.5% of scallop mantle powder. The protein and crude lipid contents of the three diets and the diet ingredients are listed in Table 1.

### 2.2. Feeding Experiment

Sea cucumbers (*A. japonicus*) with an average body weight of 3.8 ± 2.3 g were cultured in opaque plastic tanks of 250 L with a diameter of 0.26 m and height of 1.2 m. Three replications were designed for each feeding treatment with all replicates being fully randomized. Eighty sea cucumbers were cultured in each tank. During the experiment, the water was continuously aerated and maintained at 16.0 ± 1.0 °C, pH 8.0 and salinity of 30–32 psu. The sea cucumbers were fed at 9:00 am and 16:00 pm every day and the diet amount was about 5.0% of the total sea cucumber body weight in each tank. Half the volume of the water was exchanged with seawater after sand-filtering and UV treatment every day. The experiment lasted for 40 days. On days 0, 20 and 40 during the experiment, all of the sea cucumbers in each tank were weighted to calculate the specific growth rate (SGR). Six sea cucumbers were randomly selected on days 0, 15, 30 and 40. They were dissected and their intestinal tract, respiratory tree and body wall were collected, mixed and frozen at −80 °C for tissue fatty acid and amino acid compositions and intestinal gene expressions and microflora.

### 2.3. Fatty Acid and Amino Acid Analysis

Tissues of sea cucumbers collected on day 40 were homogenized, frozen-dried and then ground into fine powder. The dry sea cucumber tissue powder, scallop visceral mass powder and mantle powder were analyzed for fatty acid and amino acid compositions and *S. thunbergii* powder for fatty acid compositions. The samples were weighted and extracted for total lipids using chloroform/methanol (2:1) with 0.01% butylated hydroxytoluene (BHT) as an antioxidant and 19:0 FAME (fatty acid methyl ester) as an internal standard. The extracts were hydrolyzed using 6% KOH methanol, acidified with HCl to pH 2 and then esterified using 14% boron trifluoride–methanol. FAMEs were quantified using gas chromatography (Agilent 7890A) equipped with a DB-FFAP capillary column (30 m × 0.32 mm × 0.25 μm). The temperature programs were set as 150 °C for 1.0 min, rate of 3 °C/min to 220 °C for 33 min.

For amino acid analysis, about 20 mg samples were weighed and put into an ampoule bottle. Then, 10 mL 1 mol/L HCl was added, and the bottle was filled with nitrogen gas and then sealed. The sample in the bottle was hydrolyzed at 110 °C for 24 h, and then evaporated to dryness. Pre-column derivation of 200 μL sample was conducted by adding 100 μL trimethylamine and 100 μL phenyl isothiocyanate for 1 h, and then extracted by hexane. The filtered sample was analyzed for amino acids with a high-performance liquid chromatograph (Agilent 1100) equipped with a Venusil-AA column (4.6 × 250 mm, 5 μm). The mobile phase A was 0.1 mol/L sodium acetate with 7% acetonitrile and mobile phase B was 80% acetonitrile. Flow velocity was 1 mL/min. The wavelength was 254 nm.

### 2.4. Immune-Related Gene Expression

The expressions of immune-related genes including TLR3, AjToll, MyD88, TRAF6, p50, p105, rel, MKK36 and p38 in the intestine of *A. japonicus* collected on day 40 were analyzed. AjToll is one of the Toll-like receptor genes identified from sea cucumber *A. japonicus*, which is functionally involved in the immune responses of *A. japonicus* [11]. Total RNA from the intestine was extracted using a SPARK easy Improved Tissue/Cell RNA Kit (SparkJade, Jinan, China). The cDNA was generated by Prime Script™ RT reagent Kit (Takara, Japan). The genes were determined using qPCR performed with SYBR^®®^ Green Premix Pro Taq HS qPCR Kit (Accurate Biology, Changsha, China) in a CFX96 Touch Real-Time PCR Detection System (Bio-Rad, Hercules, CA, USA). The primers were listed in Supplementary Table S1. qPCR was run in triplicate with the reference gene using the following protocols: 30 s at 95 °C, followed by 39 cycles of 5 s at 95 °C and 30 s at 57 °C. Data were quantified using the 2^−ΔΔCT^ method.

### 2.5. Intestinal Microflora Analysis

Intestinal microflora of the sea cucumber collected on day 40 were analyzed. The sample DNA was isolated from the intestine of *A. japonicus* using the CTAB method [12]. 16S rDNA amplicon PCR was performed targeting the V3-V4 region using the primers of 341F-806R. Sequencing libraries were generated using TruSeq^®®^ DNA PCR-Free Sample Preparation Kit (Illumina, San Diego, CA, USA), and its quality was evaluated on the Qubit@ 2.0 Fluorometer (Thermo Fisher Scientific, Waltham, MA, USA) and Agilent Bioanalyzer 2100. Then, the library was sequenced on an Illumina NovaSeq platform, which generated 250 bp paired-end reads.

Paired-end reads were merged using FLASH (V1.2.7) [13]. Quality filtering on the raw tags was carried out to obtain high-quality clean tags based on the QIIME (V1.9.1) quality-controlled process [14]. Sequence analysis was performed by Uparse software (V7.0.1001) [15]. Sequences with ≥97% similarity were assigned to the same OTU. The OTU sequence was made taxonomic annotations using the Silva Database (release 138.1) [16]. 

### 2.6. Calculation and Statistical Analysis

The specific growth rate (SGR, %/d) of sea cucumber was calculated as

$$\mathrm{SGR}\left(\%/d\right)=\frac{\ln\left(\frac{W_{f}}{W_{i}}\right)}{t}\times 100.$$

In which *W_i_* and *W_f_* are the initial and final body weight (g), respectively, *t* is the duration time of the experiment (d).

The normality and homogeneity of variances of all the data were tested by Shapiro–Wilk test and Levene’s test, respectively. All data of SGRs, amino acid and amino acid compositions, and the log-transformed data of gene expressions were normally distributed and had homogeneity of variances. Their significant differences among different dietary groups were assessed by one-way ANOVA analysis followed by Turkey and Duncan’s multiple comparison tests (*p* < 0.05) with the software SPSS 22.0.

Shannon and Simpson index of the intestinal microflora were calculated using Qiime (V1.7.0). The data of the Shannon and Simpson index were not normally distributed, and thus a Kruskal–Wallis pairwise test (*p* < 0.05) was performed to assess the significant differences among dietary groups by the agricolae package in the R platform (V3.5.3). Linear discriminate analysis effect size (LEfSe) was performed to identify the potential biomarkers of microbial. The threshold on the linear discriminant analysis (LDA) score for biomarkers was 3.0. Spearman correlations between the intestinal microbes and gene expressions were conducted by the “psych” package in the R platform (V3.5.3). 

## 3. Results

### 3.1. Growth Performance of A. japonicus

**Figure 1:**
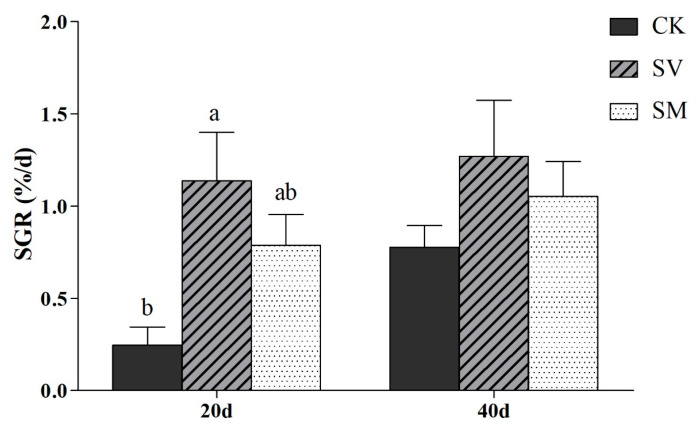
Specific growth rate (SGR, %/d) of *Apostichopus japonicus* in different dietary groups on days 20 and 40. Different letters above bars denote significant differences among different groups (*p* < 0.05). CK: control group with basal diet; SV: feeding group with supplementation of scallop visceral mass; SM: feeding group with supplementation of scallop mantle.

*A. japonicus* in CK, SV and SM dietary groups had grown to an average weight of 4.00, 4.78 and 4.45 g by day 20 and 5.19, 6.41 and 5.83 g by day 40, respectively. The SGR in the SV group was the highest, followed by the SM group, while the CK group was the lowest on day 20 and day 40 (Figure 1). The SGR of the SV group on day 20 is significantly higher than that of the CK group (*p* < 0.05), with an increase of 307%. 

### 3.2. Fatty Acid Compositions

**Figure 2:**
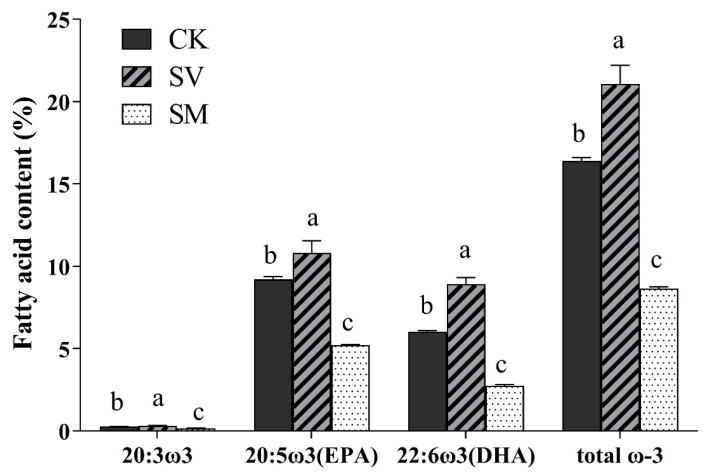

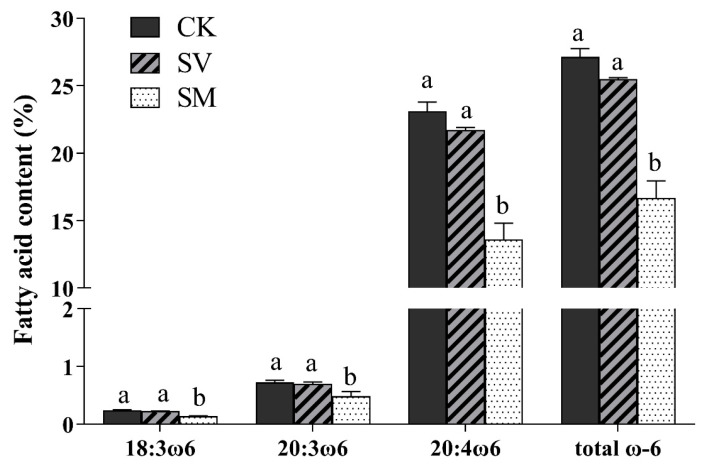
Contents of ω-3 and ω-6 fatty acid components (%) in *Apostichopus japonicus* tissue from different groups. Different letters above bars denote significant differences among different groups (*p* < 0.05). CK: control group with basal diet; SV: feeding group with supplementation of scallop visceral mass; SM: feeding group with supplementation of scallop mantle.

Fatty acid compositions of sea cucumber tissue were expressed as the percentage proportion of each fatty acid to the total amount of fatty acids (Supplementary Table S2). Sea cucumbers in the CK and SV groups had significantly higher proportions of polyunsaturated fatty acid (PUFA) than those in the SM group, while the latter had a higher proportion of saturated fatty acid (SFA) and monounsaturated fatty acid (MUFA) (*p* < 0.05). Sea cucumbers in the SV group had the highest proportion of total ω-3 fatty acid, followed by group CK, and then group SM (Figure 2). Specifically, 20:3ω3, 20:5ω3 (EPA) and 22:6ω3 (DHA) all exhibited the highest proportion in the SV group, which were 18.5, 17.5 and 48.3% higher than those in the CK group, respectively (Figure 2). In contrast, 18:3ω6, 20:3ω6, 20:4ω6 and total ω-6 fatty acids displayed comparable proportions in groups CK and SV, but higher than those of the SM group (*p* < 0.05). The ratio of ω-3/ω-6 fatty acids in the SV group reached up to 0.83, and it was significantly higher than 0.6 and 0.52 in the CK and SM groups, respectively. 

### 3.3. Amino Acid Compositions

Amino acid compositions of sea cucumber tissue from different dietary groups are shown in Supplementary Table S3. The mass percentage of total amino acids summed up to 41.16 ± 2.32%, 41.38 ± 1.99% and 41.19 ± 1.41% in the CK, SV and SM groups, respectively. Essential amino acids accounted for 30 ± 1, 30 ± 1 and 29 ± 1% of the total amino acids in the CK, SV and SM groups, respectively. There are no significant differences for most of the amino acid components, total amino acid, total essential amino acid and total nonessential amino acid contents among three dietary groups, except histidine. Histidine contents in the CK and SV groups were significantly higher than those in the SM group (*p* < 0.05). 

### 3.4. Immune-Related Gene Expressions

**Figure 3:**
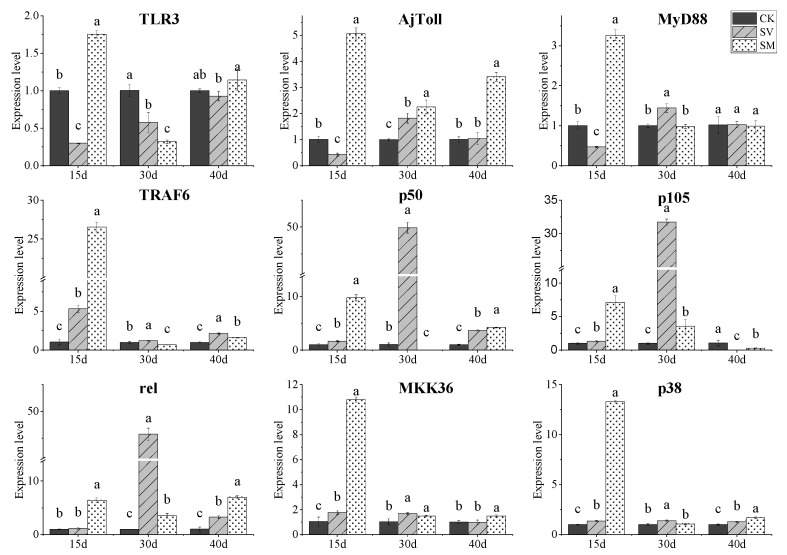
Expression levels of immune-related genes in intestine of *Apostichopus japonicus* from CK, SV and SM dietary groups on day 15, 30 and 40, respectively. Different lowercase letters denote significant differences (*p* < 0.05). CK: control group; SV: dietary supplementation with 3.5% scallop visceral mass powder; SM: dietary supplementation with 3.5% scallop mantle powder.

The expression levels of immune-related genes including TLR3, AjToll, MyD88, TRAF6, p50, p105, rel, MKK36 and p38 in the intestine of *A. japonicus* were determined (Figure 3). The expression levels of most of these genes in the SV and SM groups were higher than those in the CK group (*p* < 0.05). For the SM group, the expression levels of these genes on day 15, except TLR3, increased by 2.3–24.6 times compared with those in the CK group. The expression of p50, p105 and rel genes in the SV group on day 30 increased by 47.1, 30.6 and 44.8 times compared with those in the CK group, respectively. The expression levels of TRAF6, MKK36 and p38 genes in the SV group on day 15 were 4.1, 0.7 and 0.4 times higher than those in the CK group. 

### 3.5. Intestinal Microbial Community

#### 3.5.1. Microbial Abundance and Composition

**Figure 4:**
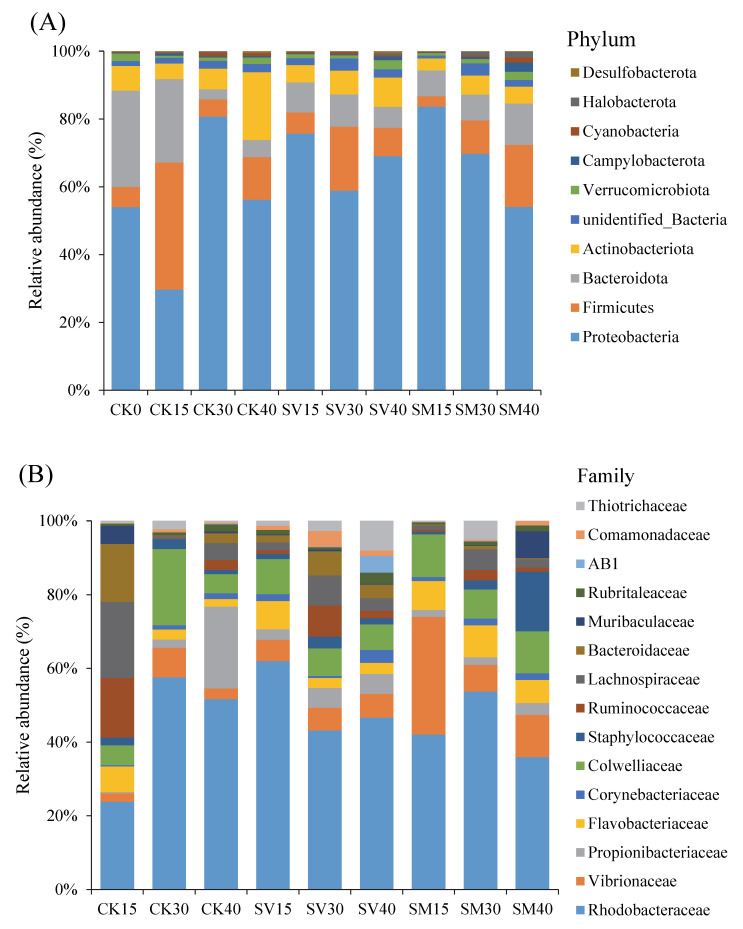

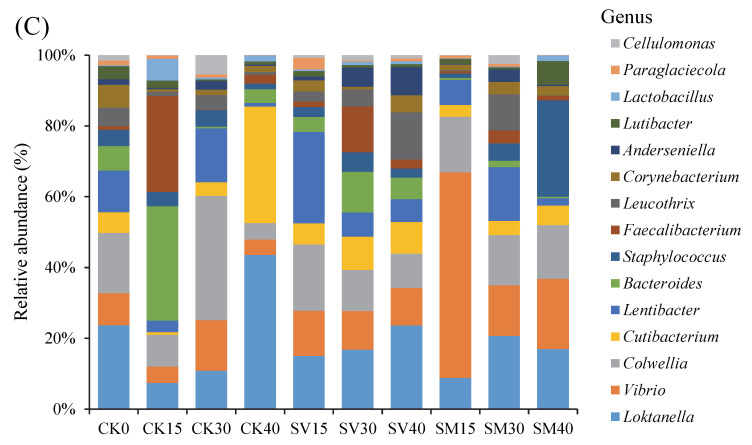
Composition and relative abundance (%) of microbial community in intestine of *Apostichopus japonicus* from each group at (**A**) phylum, (**B**) family and (**C**) genus level, respectively. CK: control group with basal diet; SV: feeding group with supplementation of scallop visceral mass; SM: feeding group with supplementation of scallop mantle; 0, 15, 30 and 40 denote 0, 15, 30 and 40 days, respectively.

Relative abundances of the top 10 phylum, class and top 15 order, family and genus microbes were presented in Figure 4 and Supplementary Figure S1. Phylum Proteobacteria was the dominant abundant microbe in the intestine of *A. japonicus*, accounting for 29.1–80.3% of the entire community. Then, it was followed by Bacteroidota and Firmicutes, accounting for 3.0–36.7% and 2.8–27.1%, respectively. These three phyla accumulated to 71.8–90.6% of the entire community. The most four abundant microbial classes were Alphaproteobacteria, Gammaproteobacteria, Bacteroidia and Clostridia, accounting for 18.3–51.0, 10.8–42.4, 2.7–27.1 and 1.1–30.5% of the total microbe, respectively. At the family level, Rhodobacteraceae was the most abundant, accounting for 16.1–45.4%, followed by Vibrionaceae, Propionibacteriaceae, Flavobacteriaceae, etc. The top five most abundant genera were *Loktanella*, *Vibrio*, *Colwellia*, *Cutibacterium* and *Lentibacter*. 

#### 3.5.2. Alpha Diversity of Microbial Community

**Figure 5:**
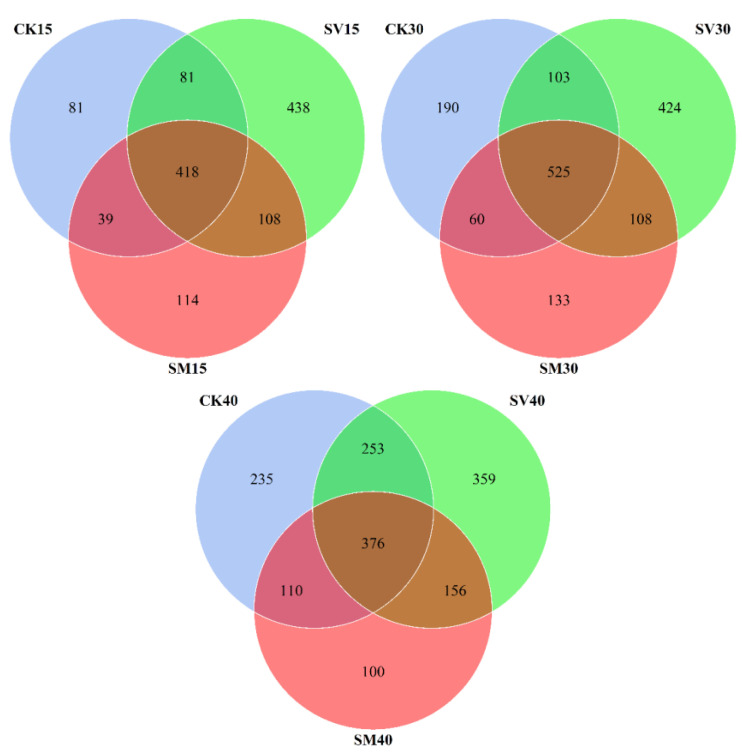
Venn diagrams of OTUs in the intestine of *Apostichopus japonicus* from CK, SV and SM dietary groups on days 15, 30 and 40, respectively. CK: control group with basal diet; SV: feeding group with supplementation of scallop visceral mass; SM: feeding group with supplementation of scallop mantle.

**Figure 6:**
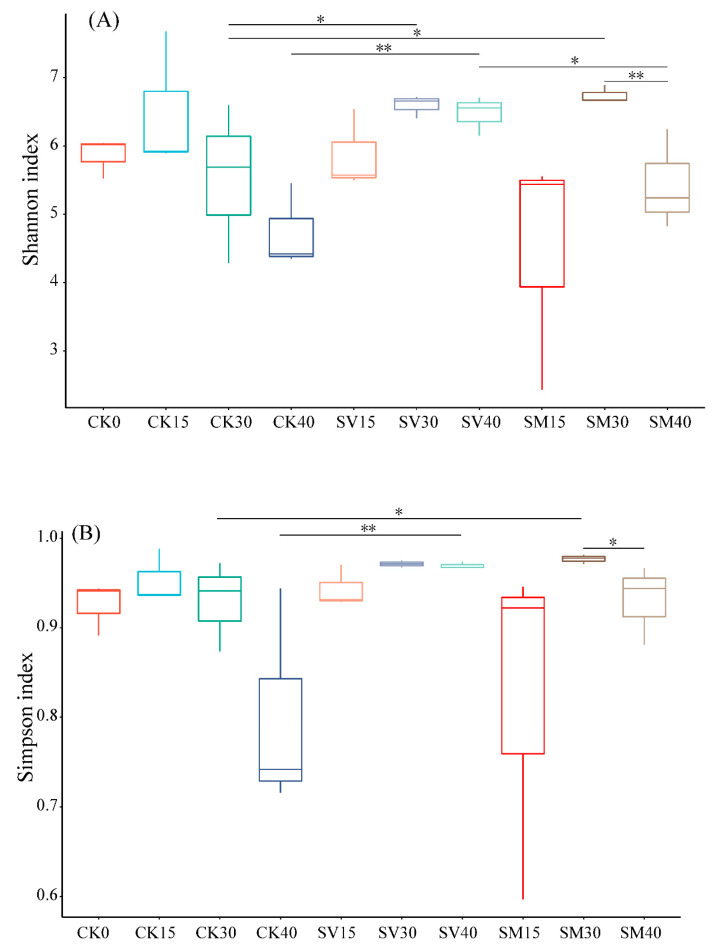
Alpha diversity of microbial community in the intestine of *Apostichopus japonicus* from groups CK, SV and SM on days 0, 15, 30 and 40. (**A**) Shannon index, (**B**) Simpson index. * *p* < 0.05, ** *p* < 0.01. CK: control group with basal diet; SV: feeding group with supplementation of scallop visceral mass; SM: feeding group with supplementation of scallop mantle.

Venn diagram (Figure 5) showed that the intestine microbe in the SV group exhibited the largest number of unique OTUs during the whole experiment process, while the microbe in the SM group displayed the smallest number of unique OTUs. The Shannon diversity index of the microbial community in the SV group on days 30 and 40 and that in the SM group on day 30 were significantly higher than the Shannon index of the CK group on the same days (*p* < 0.05) (Figure 6). The Simpson index in the SV group on day 40 and in the SM group on day 30 were also significantly higher than the Simpson index of the CK group on the same days (*p* < 0.05).

#### 3.5.3. LEfSe Analysis

**Figure 7:**
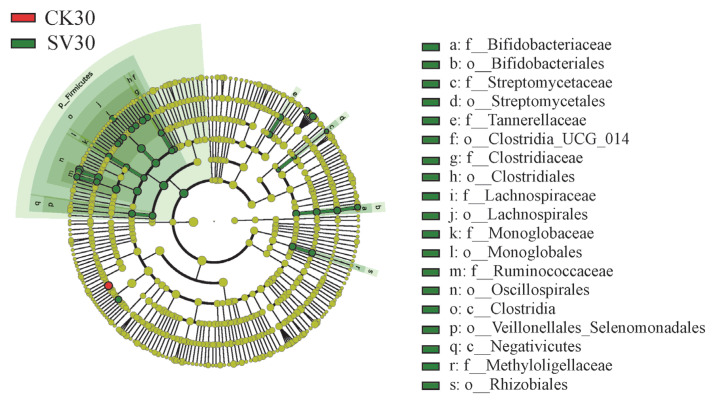

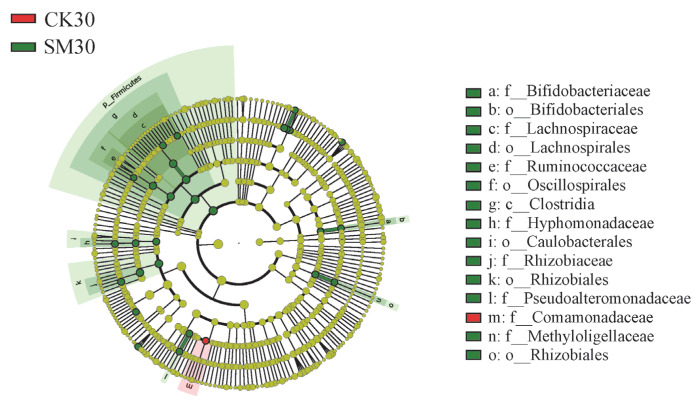
Cladogram of the microbial communities in the intestines of *Apostichopus japonicus* from different dietary groups on day 30. The color mode means differentially abundant taxa identified as biomarkers in different treatments. The six rings of the cladogram from inner to outside stand for phylum, class, order, family, genus and species. CK: control group with basal diet; SV: feeding group with supplementation of scallop visceral mass; SM: feeding group with supplementation of scallop mantle.

The LEfSe was used to identify differentially abundant taxa (biomarkers) among different treatments, whose abundances in a certain treatment are significantly higher than those in other treatments (Figure 7, Supplementary Figure S2). On day 15, the biomarkers in the CK group were more than those in the SV and SM groups, while on days 30 and 40, the biomarkers were mainly found in the SV and SM groups. On day 30, family Bifidobacteriaceae, Streptomycetaceae, Tannerellaceae, Clostridiaceae, Lachnospiraceae, Monoglobaceae, Ruminococcaceae, Methyloligellaceae and order Rhizobiales were identified as biomarkers in the SV group, while family Bifidobacteriaceae, Lachnospiraceae, Ruminococcaceae, Hyphomonadaceae, Rhizobiaceae, Pseudoalteromonadaceae, Comamonadaceae and Methyloligellaceae were biomarkers in the SM group. On day 40, biomarkers in the SV group included the family Nocardioidaceae, Granulosicoccaceae and Holomonadaceae and order Oceanospirillales and Rhizobiales. Biomarkers in the SM group included the family Acrobacteraceae, Comamonadaceae, Pseudomonadaceae, Vibrionaceae, etc. 

## 4. Discussion

Scallop visceral mass and mantle are generally considered as inedible portions of aquatic products. However, they are rich in protein, amino acids, fatty acids and other nutrient substances [9,17], which are potential sources for the feed supplement of precious marine products. In this case, we turned our attention towards the effects of dietary scallop visceral mass and mantle on growth, amino acid and fatty acid profiles, immune responses and intestinal microbiota of sea cucumber (*A. japonicus*). Growth performance is one of the most important indices to evaluate the effects of sea cucumber aquaculture. Our results show that the dietary supplementation of scallop visceral mass significantly promoted the growth rate of *A. japonicus* within 20 days, with the SGR increased by 307% on day 20 compared with that in the CK group. It should be noted that the average SGR of day 40 in group SV was also 62.8% higher than that in the CK group, although no significant difference was detected. The lack of significant difference probably suggests a less significant stimulation effect of scallop visceral mass at a later stage of the raring, or because of the large standard deviation of the SGRs. Larger standard deviations of the SGRs were observed on day 40, which probably resulted from the increase in unpredicted influencing factors, the accumulation of variances with time, and also from the uncertainty of living animals. But, a higher SGR average of group SV on day 40 might still suggest a beneficial effect of scallop visceral mass on the growth of juvenile *A. japonicus.*

Fatty acids, especially highly unsaturated fatty acids (HUFAs), have been reported to play important roles in the physiology and reproductive processes of both plants and animals [18,19]. Tissue fatty acid composition is an important index of the metabolism and growth of sea cucumbers [20]. In this study, the results showed that dietary supplementation of scallop visceral mass significantly increased the contents of ω-3 fatty acids including 20:3ω3, EPA and DHA, as well as ω-3/ω-6 ratio. ω-3 fatty acid is essential to the growth and reproduction of sea cucumber. It was reported that different levels of EPA and DHA in the diet might have altered the reproductive strategies of *Parastichopus californicus* [21,22], and suitable supplement level of ω-3 HUFAs in diet improved the growth rate and immunity of *A. japonicus* [22]. Therefore, higher ω-3 fatty acid content in *A. japonicus* tissue indicates the beneficial effect of scallop visceral mass on the health of sea cucumbers. On the other hand, ω-3 PUFAs such as EPA and DHA bring more nutritional benefits to human health, and a diet with a high ratio of ω-3/ω-6 fatty acids is more desirable in reducing the risk of many chronic diseases, such as cardiovascular disease, cancer and inflammatory [23]. Therefore, higher ω-3 fatty content and ω-3/ω-6 ratio also suggest higher commercial value of *A. japonicus*.

The increase in tissue ω-3 fatty acids in the SV group likely results from the extremely high content of total lipid, as well as ω-3 fatty acids in the scallop visceral mass (Table 1). In addition, the protein contents of scallop visceral mass and mantle approach that of fish powder (about 70%) [24] and far exceed that of algae (Table 1). Therefore, scallop visceral mass and mantle could be used as the protein source substituting fish powder in the sea cucumber diet, and increasing the ω-3 fatty acid content of sea cucumber tissue at the same time.

Previous studies on sea cucumber culture have indicated that dietary supplementation of probiotics and biologically active substances could regulate intestinal microbiota and immunity [25,26,27]. Hence, we further investigated the effect of dietary supplementation of scallop visceral mass and mantle on the immune-related gene expressions and intestine microbial community of *A. japonicus*. Sea cucumbers are invertebrates that lack adaptive immune responses, and their defense mechanisms mainly rely on the nonspecific immune system of pattern recognition receptors (PRRs) and signal transduction [28]. Toll-like receptors (TLRs) are well-characterized among the various types of PRRs in *A. japonicus*, which could specifically recognize conserved molecular structures and activate the immune system [29]. TLRs can recruit adaptor molecules MyD88 and TRAF6 for signal transduction to activate nuclear factor-kappa B (NF-κB) and mitogen-activated protein kinases (MAPKs) pathways [30,31,32]. Here, it was found that the expressions of some genes related to the Toll-like receptor signal transduction in the nonspecific immune system were up-regulated by dietary supplementation of scallop visceral mass and mantle, including AjToll, MyD88 and TRAF6 (two key adaptor molecules); p50, p105 and rel (three NF-κB proteins); and MKK36 and p38 (two MAPK proteins). These results suggest a promising effect of dietary scallop visceral mass and mantle on enhancing the immune defense of *A. japonicus*. Additionally, it was shown that the benefit of dietary scallop visceral mass and mantle for the immune system has a time effect. For the SM group, the up-regulation of these immune-related genes showed the most remarkable effect on day 15, while the SV group exhibited the best immunostimulatory activity on day 30, suggesting different action modes of scallop visceral mass and mantle on immunity regulation of the sea cucumber.

Furthermore, dietary supplementation of scallop viscera and mantle has also manipulated the diversity of the microbial community in the intestine of *A. japonicus*. Dietary scallop visceral mass markedly increased the specific OTUs of intestinal microbes during the whole process (Figure 5), suggesting higher microbial diversity and more potential functions of the microbial community. The microbial diversity index and the number of biomarkers were significantly increased with supplementation of scallop visceral mass and mantle (Figure 6 and Figure 7), especially on day 30, when the abundances of Bifidobacteriaceae, Streptomycetaceae, Clostridiaceae, Lachnospiraceae, Monoglobaceae and Rhizobiales markedly increased.

Family Bifidobacteriaceae, Streptomycetaceae, and Clostridiaceae in the gut of *A. japonicus* were mainly composed of the genus *Bifidobacterium*, *Streptomyces* and *Clostridium*, respectively. *Bifidobacterium* is one of the best-known probiotic bacteria, exhibiting antagonistic activities against microbial pathogens, immunomodulatory, antimutagenic and anticarcinogenic activities, and the effects of prevention and cure of pathogen-induced diarrheas [33,34,35,36]. *Streptomyces* could produce many kinds of metabolites that have been used as antibiotics and the primers of drugs with strong inhibition of bacteria and pathogens [37,38]. *Streptomyces* has been used in aquaculture and demonstrates the potential of bioremediation and improving animal growth and water quality [39,40]. *Clostridium* is one of the richest bacterial clusters in the intestine of humans and animals. *Clostridium* species have been reported to attenuate inflammation and maintain intestinal health via their cellular components and metabolites including butyrate, secondary bile acids and indolepropionic acid [41]. It has been used in aquaculture and exhibited beneficial effects of promoting growth performance, immune response and digestive enzyme activities [42]. *Rhizobiales* has been found in the intestines of zebrafish and sharks and is associated with nitrogen fixation [43,44]. The existence of *Rhizobiales* could alleviate nitrogen limitation through nitrogen fixation, producing bacterial nifH protein and enhancing the growth of the colony [43].

It was shown that supplementation of scallop visceral mass and mantle increased the microbial diversity and the abundance of beneficial microbes, which might facilitate the establishment of a healthier microbial ecosystem in the intestine of *A. japonicus*. Existing studies show that the dietary supplementation of probiotics might improve the innate immunity of sea cucumbers [45,46]. Correlations between immune-related gene expressions and major microbial biomarker families in the intestine (Supplementary Figure S3) might also suggest that the enhancement of the immune response of *A. japonicus* was associated with the optimization of microbial community with dietary supplementation of scallop visceral mass and mantle.

## 5. Conclusions

The effects of dietary supplementation of scallop visceral mass and mantle on the growth, immunity and intestinal microbial community of *A. japonicus* have been investigated. The results show that dietary supplementation of scallop visceral mass increased the specific growth rate of sea cucumber by 3 times on day 20, and raised the ω-3 fatty acid contents of sea cucumber tissue including 20:3ω3, EPA and DHA due to extremely high content of fatty acid in the visceral mass. Additionally, dietary supplementation of scallop visceral mass and mantle both promoted the non-specific immunity and optimized the composition of intestinal microflora of *A. japonicus* by increasing the microbial diversity and beneficial taxa abundance including *Bifidobacteriaceae, Streptomycetaceae, Clostridiaceae* and *Rhizobiales*. This study demonstrated for the first time the comprehensive effect of dietary supplementation of the scallop visceral mass on sea cucumber culture. It revealed the promising application of scallop visceral mass and mantle in the feed of sea cucumber and the potential of high-value utilization of this scallop “waste”.

## Figures and Tables

**Table 1:** Protein and crude lipid contents (%) of the diet for CK, SV and SM groups and the diet ingredients.

	Diet-CK	Diet-SV	Diet-SM	Scallop Visceral Mass	Scallop Mantle	*S. thunbergii*	Sea Mud
Protein	9.2 ± 0.3	10.7 ± 0.1	11.2 ± 0.01	53.2	66.2	19.0	2.7
Lipid	0.98 ± 0.2	1.4 ± 0.1	1.0 ± 0.2	12.5	2.4	2.0	0.3

## Data Availability

The data supporting the findings of this study are available from the corresponding author upon request.

## Supplementary Materials

The following supporting information can be downloaded at: https://www.mdpi.com/article/10.3390/biology12091239/s1, Table S1: primers for gene expression; Table S2: fatty acid composition in tissue of sea cucumber and feed ingredients (%, dry mass); Table S3: amino acid composition in tissue of sea cucumber and feed ingredients (%); Figure S1: composition and relative abundance (%) of microbial community in each group at (A) class and (B) order levels, respectively; Figure S2: cladogram of the microbial communities in different dietary supplement groups on days 15 and 40 by a linear discriminant analysis (LDA) with a threshold of 3. The color mode means differentially abundant taxa identified as biomarkers in different treatments. The six rings of the cladogram from inner to outside stand for phylum, class, order, family, genus and species; Figure S3: correlations between the immune-related genes and top 20 families and major biomarker families of microbial community in intestine of the *A. japonicus*. (* *p* < 0.05, ** *p* < 0.01).
