# Supplementary material for: Effects of Scallop Visceral Mass and Mantle as Dietary Supplements on the Growth, Immune Response and Intestinal Microflora of Juvenile Sea Cucumber Apostichopus japonicus
Source: Biology (Basel). 2023 Sep 14;12(9):1239. doi: 10.3390/biology12091239 (PMC10525178; doi:10.3390/biology12091239)
Supplement: Supplementary file 1 [file biology-12-01239-s001.zip › biology-2586353-supplementary.pdf]

Supplementary material

Table S1. Primers for gene expression.

Target genes	Primers	Sequence
cytb*	F	TGAGCCGCAACAGTAATC
	R	AAGGGAAAAGGAAGTGAAAG
TLR3	F	AAATACGAACACGATGTAGCGA
	R	TTCTTTTCAATGGACGAGGC
AjToll	F	ACGAAAGCGATTTAGCC
	R	GAGCCCGTGGTGAGATG
MyD88	F	TTTAGCCTTAGGACCGACCG
	R	GCCTCTTTTACTCCCCCAATTA
TRAF6	F	TGGGAAGCAAATCGCAAA
	R	CGACTACCACCTTAGGACACGT
p50	F	TCCTATCGGTCTGAATCTTCCAA
	R	TTTCTTCCCTTTCTGGCTATGTTC
p105	F	GCAACACACCCCTCCATCTT
	R	TCTTCTTCGCTAACGTCACACC
rel	F	TGAAGGTGGTATGCGTCTGG
	R	TTGGGCTGCTCGGTTATG
MKK36	F	CCGAGGAGAAAGGATCAAGAGA
	R	TTATGACAGGGTAGCCACACAA
p38	F	ACATAGACATAAATCCAATCGGC
	R	AAGGTGAAAATACATCCAACAGG

*Note: The cytb gene was selected as the reference gene.

Table S2. Fatty acid composition in tissue of sea cucumber and feed ingredients (%, dry mass).

Fatty acids	CK	SV	SM	Scallop visceral mass	Scallop mantle	*S.thunbergii*
C12:0	ND	ND	ND	0.25	0.10	0.20

C13:0	ND	ND	ND	0.00	0.00	0.50
C14:0	1.75±0.09b	1.53±0.27b	2.29±0.39a	6.34	ND	6.68
C14:1	0.56±0.17b	0.58±0.04b	0.81±0.28a	0.04	0.20	0.00
C15:0	0.18±0.01b	0.17±0.05b	0.32±0.03a	0.56	1.39	0.41
C16:0	11.79±1.19b	9.76±0.89c	14.81±0.7a	22.01	28.26	31.47
C16:1	8.77±0.27b	9.11±0.95b	11.36±1.54a	19.01	5.46	5.23
C17:0	1.09±0.02b	0.98±0.09c	1.83±0.19a	0.67	2.27	0.22
C17:1	0.36±0.04	0.36±0.04	0.44±0.04	0.00	0.00	0.18
C18:0	7.15±0.27b	7.36±0.08b	10.78±0.2a	4.06	8.78	1.15
C18:1	10.08±0.42b	8.84±0.66c	12.82±1.43a	6.09	4.50	6.85
C18:2ω6	3.06±0.08a	2.82±0.21b	2.47±0.15b	3.03	1.77	2.48
C18:3ω6	0.23±0.00a	0.23±0.02a	0.13±0.00b	0.35	0.11	0.21
C18:3ω3	0.9±0.05a	0.99±0.08a	0.52±0.08b	0.10	0.20	1.56
C20:0	1.79±0.05b	1.77±0.07b	2.54±0.2a	0.92	0.69	0.45
C20:1ω9	5.81±0.41b	5.52±0.34b	7.84±0.38a	0.61	1.21	2.64
C20:2	1.73±0.12a	1.86±0.1a	1.41±0.13b	0.30	0.41	ND
C20:3ω6	0.72±0.05a	0.7±0.14a	0.49±0.03b	0.21	0.23	0.33
C21:0	0.79±0.09a	0.83±0.28ab	1.19±0.25b	0.00	0.00	ND
C20:4ω6	23.12±0.32a	21.73±2.1a	13.6±1.96b	0.00	0.00	6.27
C20:3ω3	0.27±0.02b	0.32±0.03a	0.16±0.04c	0.31	0.55	ND
C20:5ω3EPA	9.21±1.26b	10.82±0.04a	5.22±0.64c	20.65	16.31	13.43
C22:0	1.84±0.06b	1.81±0.1b	3.03±0.39a	0.09	0.54	19.73
C22:1ω9	1.1±0.16b	0.98±0.04b	2.24±0.58a	0.15	0.23	ND
C22:2	0.85±0.1	1.18±0	ND	0.12	0.08	ND
C23:0	0.6±0.08	0.59±0.03	0.65±0.31	0.08	0.14	ND
C24:0	0.21±0.03b	0.25±0.01b	0.32±0.07a	0.15	0.55	ND
C22:6ω3DHA	6.01±0.71b	8.91±0.15a	2.74±0.26c	13.92	26.04	ND
SFA	27.2±0.94b	25.04±1.14c	37.75±0.64a	34.87	42.63	60.11
MUFA	26.68±1.27b	25.39±1.4b	35.51±2.96a	25.90	11.58	14.91
PUFA	46.11±2.05a	49.57±2.48a	26.74±3.03b	38.99	45.69	24.28
ω-3	16.39±1.99b	21.04±0.19a	8.65±0.95c	34.97	43.09	14.98
ω-6	27.14±0.19a	25.48±2.2a	16.69±1.95b	3.59	2.11	9.30
ω-3/ω-6	0.6±0.08b	0.83±0.06a	0.52±0.01b	9.73	20.43	1.61

ND: not detected

Different letter denote significant differences between data by one-way ANOVA analysis ($p<0.05$).

Table S3. Amino acid composition in tissue of sea cucumber and feed ingredients (%)

Amino acid	CK	SV	SM	scallop visceral mass	scallop mantal
Asp	4.38±0.24	4.36±0.19	4.31±0.14	3.61	4.28
Thr	2.16±0.15	2.13±0.1	2.1±0.09	1.51	1.93
Ser	2.18±0.09	2.15±0.14	2.06±0.15	1.26	1.47

Glu	6.81±0.4	6.94±0.38	6.91±0.14	5.11	7.58
Gly	4.64±0.47	4.59±0.13	4.84±0.16	4.25	7.39
Ala	2.39±0.16	2.41±0.1	2.42±0.15	1.94	2.81
Cys	0.24±0.05	0.32±0.06	0.25±0.08	0.62	0.2
Val	1.97±0.12	1.97±0.14	1.96±0.11	1.75	2.43
Met	0.11±0.03	0.13±0.05	0.2±0.18	0.21	0.63
Ile	1.64±0.09	1.64±0.13	1.65±0.17	1.5	2.17
Leu	2.48±0.15	2.48±0.17	2.4±0.11	2.3	3.27
Tyr	0.99±0.14	0.67±0.57	1.02±0.06	0.84	1.46
Phe	1.69±0.08	1.93±0.42	1.62±0.06	1.3	1.73
His	1.62±0.02[a]	1.66±0.12[a]	1.45±0.08[b]	1.17	1.28
Lys	2.15±0.07	2.2±0.12	2.14±0.14	2.89	3.17
Arg	3.01±0.17	3.04±0.15	3.12±0.19	1.46	1.59
Pro	2.69±0.27	2.76±0.09	2.74±0.18	1.1	2
∑AA	41.16±2.32	41.38±1.99	41.19±1.41	32.82	45.39
∑EAA	12.2±0.64	12.47±0.37	12.07±0.52	11.46	15.34
∑NEAA	28.95±1.72	28.91±1.76	29.12±0.96	21.36	30.05
∑EAA/∑NEAA	0.42±0.01	0.43±0.02	0.41±0.01	0.54	0.51
∑EAA/∑AA	0.3±0.01	0.3±0.01	0.29±0.01	0.35	0.34

AA: amino acid; EAA: essential amino acid; NEAA: non-essential amino acid;

(A)

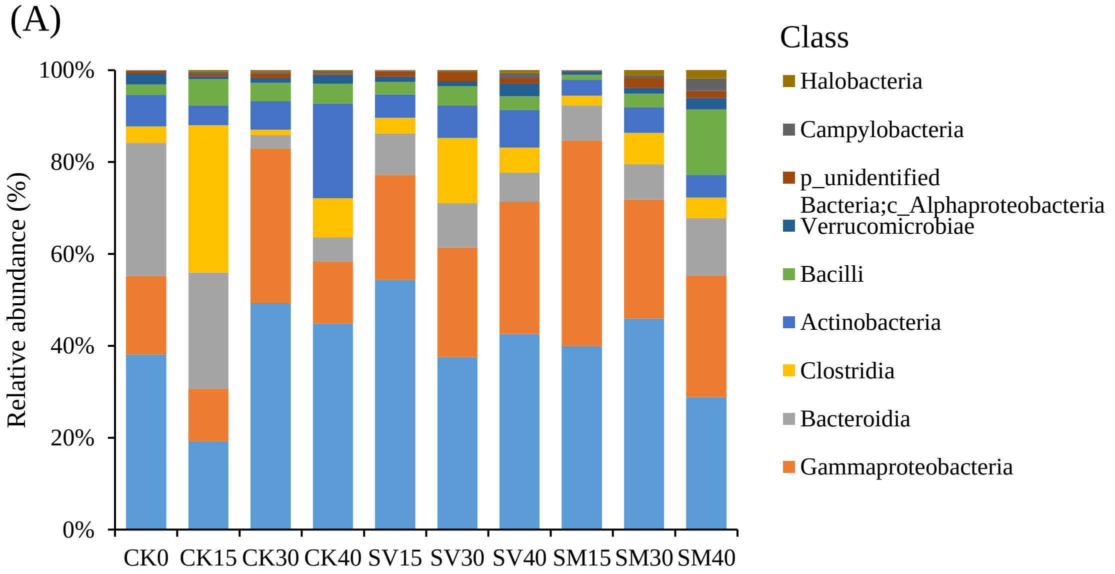

(B)

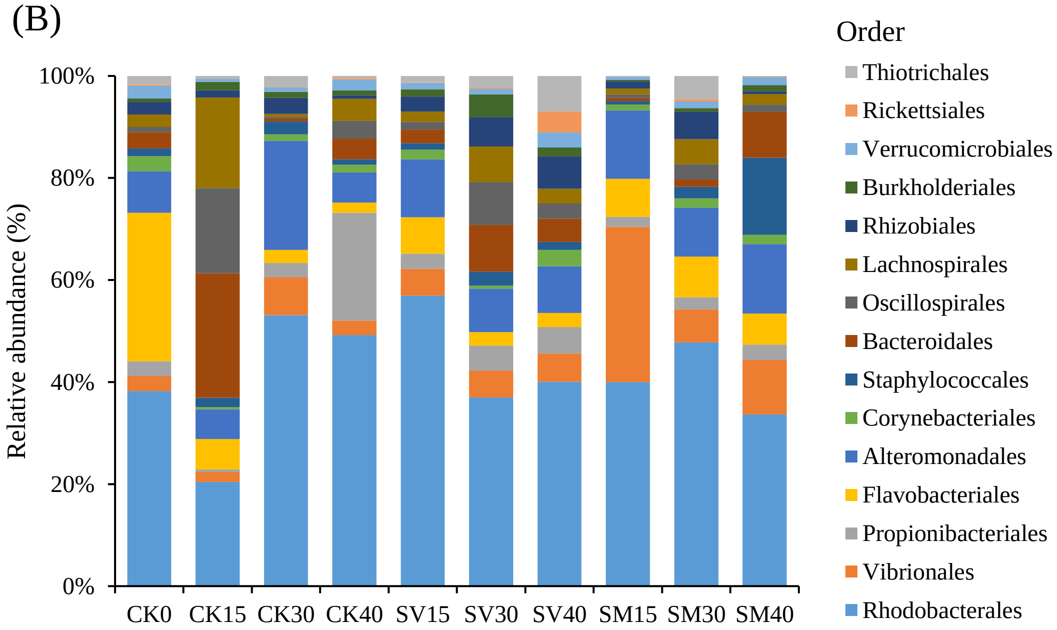

Figure S1 Composition and relative abundance (%) of microbial community in intestine of *A. japonicus* in each group at (A) Class and (B) Order levels, respectively. CK: control group with basal diet; SV: feeding group with supplementation of scallop visceral mass; SM: feeding group with supplementation of scallop mantle. 0, 15, 30 and 40 denote 0, 15, 30 and 40 days, respectively.

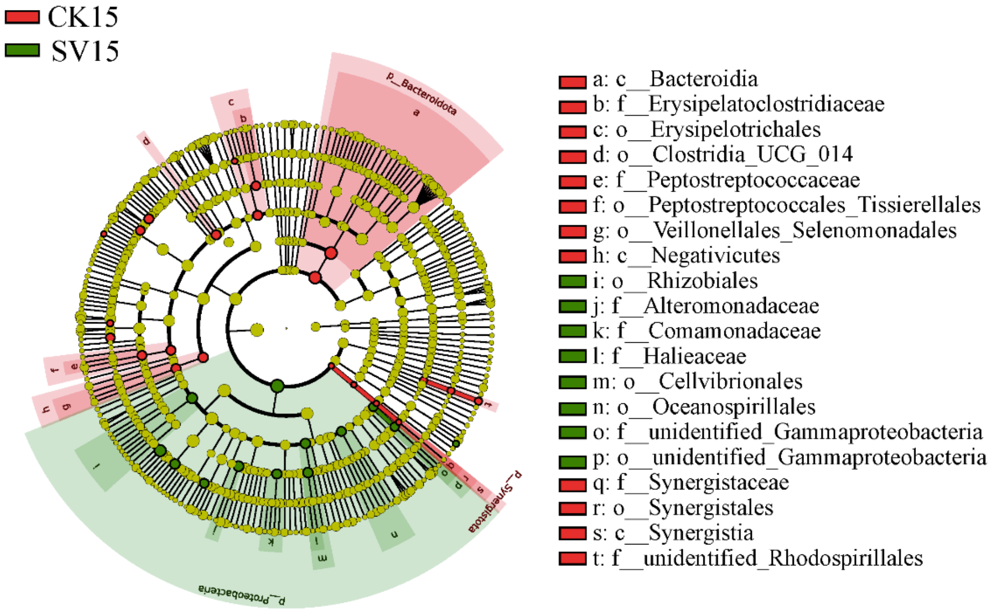
CK15
SV15
p__Bacteroidota
p__Proteobacteria
p__Synergistota
a: c__Bacteroidia
b: f__Erysipelatoclostridiaceae
c: o__Erysipelotrichales
d: o__Clostridia_UCG_014
e: f__Peptostreptococcaceae
f: o__Peptostreptococcales_Tissierellales
g: o__Veillonellales_Selenomonadales
h: c__Negativicutes
i: o__Rhizobiales
j: f__Alteromonadaceae
k: f__Comamonadaceae
l: f__Halieaceae
m: o__Cellvibrionales
n: o__Oceanospirillales
o: f__unidentified_Gammaproteobacteria
p: o__unidentified_Gammaproteobacteria
q: f__Synergistaceae
r: o__Synergistales
s: c__Synergistia
t: f__unidentified_Rhodospirillales

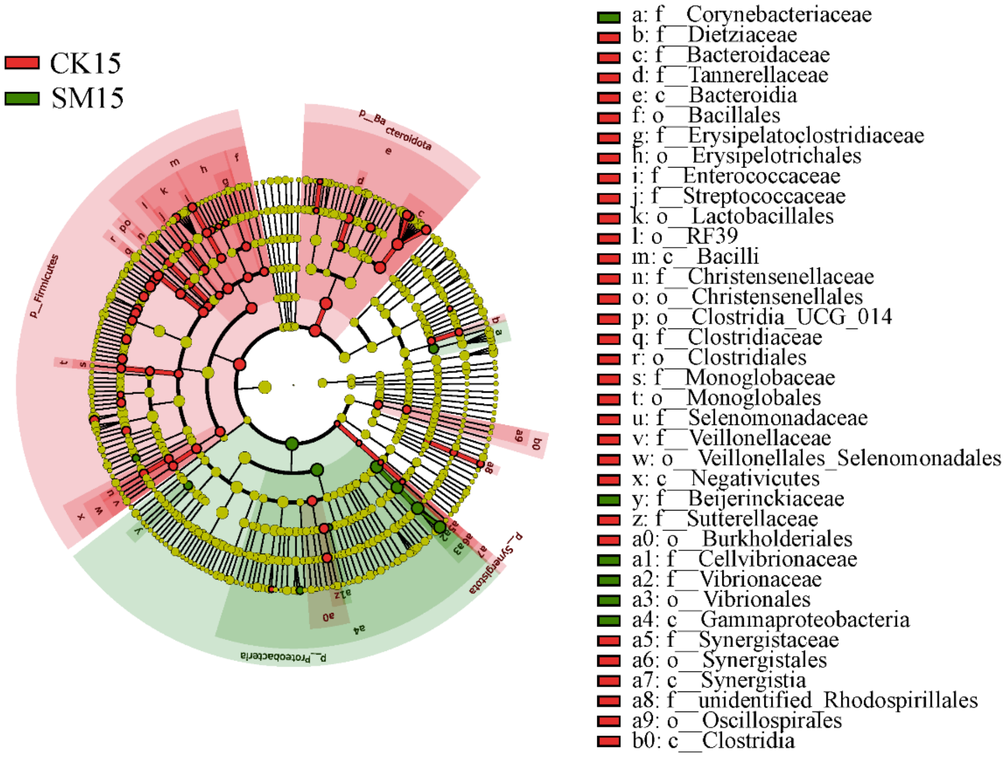
CK15
SM15
p__Bacteroidota
p__Firmicutes
p__Proteobacteria
p__Synergistota
a: f__Corynebacteriaceae
b: f__Dietziaceae
c: f__Bacteroidaceae
d: f__Tannerellaceae
e: c__Bacteroidia
f: o__Bacillales
g: f__Erysipelatoclostridiaceae
h: o__Erysipelotrichales
i: f__Enterococcaceae
j: f__Streptococcaceae
k: o__Lactobacillales
l: o__RF39
m: c__Bacilli
n: f__Christensenellaceae
o: o__Christensenellales
p: o__Clostridia_UCG_014
q: f__Clostridiaceae
r: o__Clostridiales
s: f__Monoglobaceae
t: o__Monoglobales
u: f__Selenomonadaceae
v: f__Veillonellaceae
w: o__Veillonellales_Selenomonadales
x: c__Negativicutes
y: f__Beijerinckiaceae
z: f__Sutterellaceae
a0: o__Burkholderiales
a1: f__Cellvibrionaceae
a2: f__Vibrionaceae
a3: o__Vibrionales
a4: c__Gammaproteobacteria
a5: f__Synergistaceae
a6: o__Synergistales
a7: c__Synergistia
a8: f__unidentified_Rhodospirillales
a9: o__Oscillospirales
b0: c__Clostridia

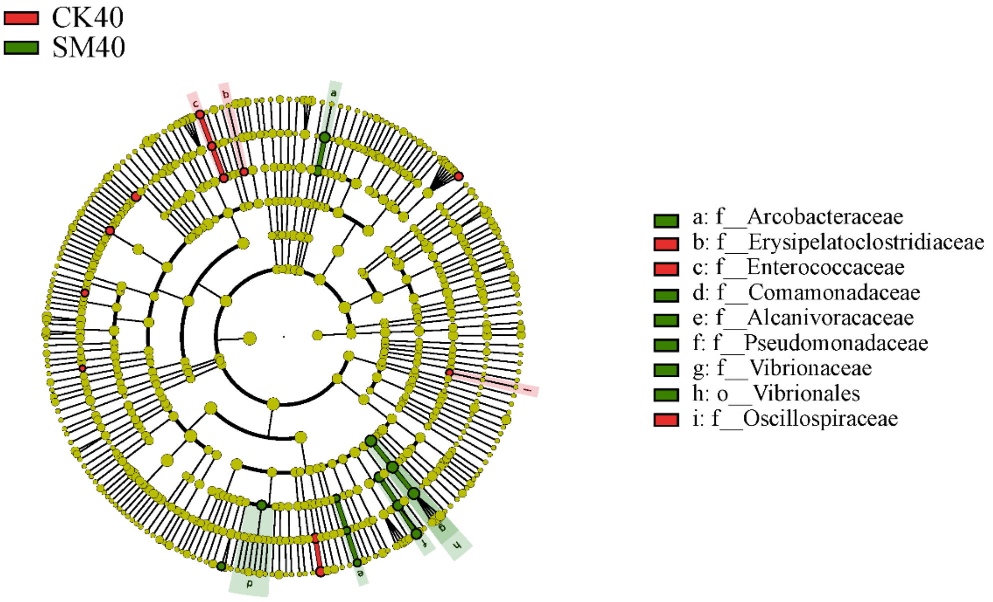

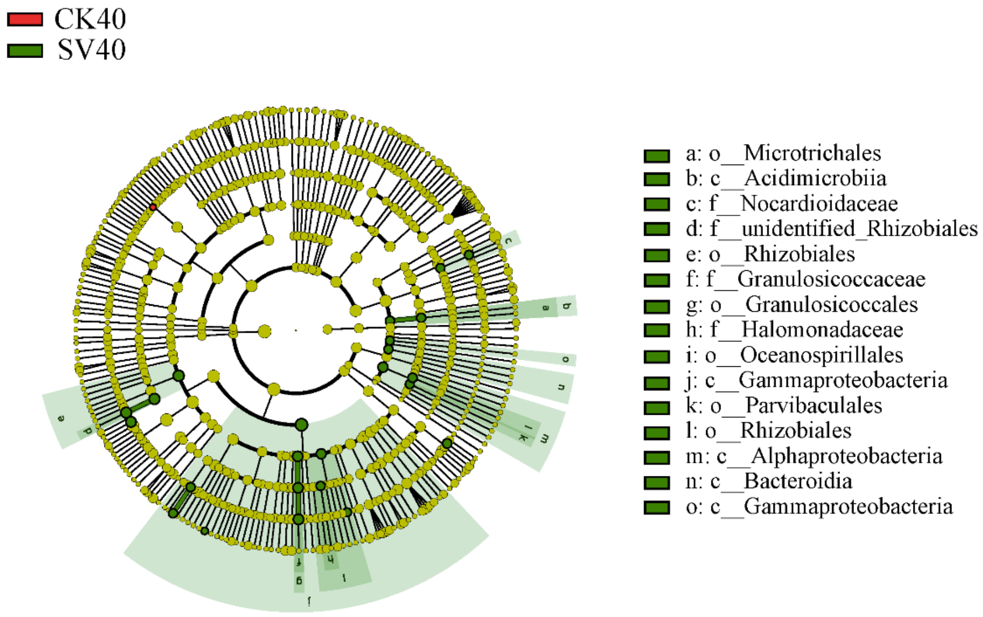

Figure S2 Cladogram of the microbial communities in different dietary supplement groups on days 15 and 40 by a linear discriminant analysis (LDA) with a threshold of 3. The color mode means differentially abundant taxa identified as biomarkers in different treatments. The six rings of the cladogram from inner to outside stand for phylum, class, order, family, genus and species.

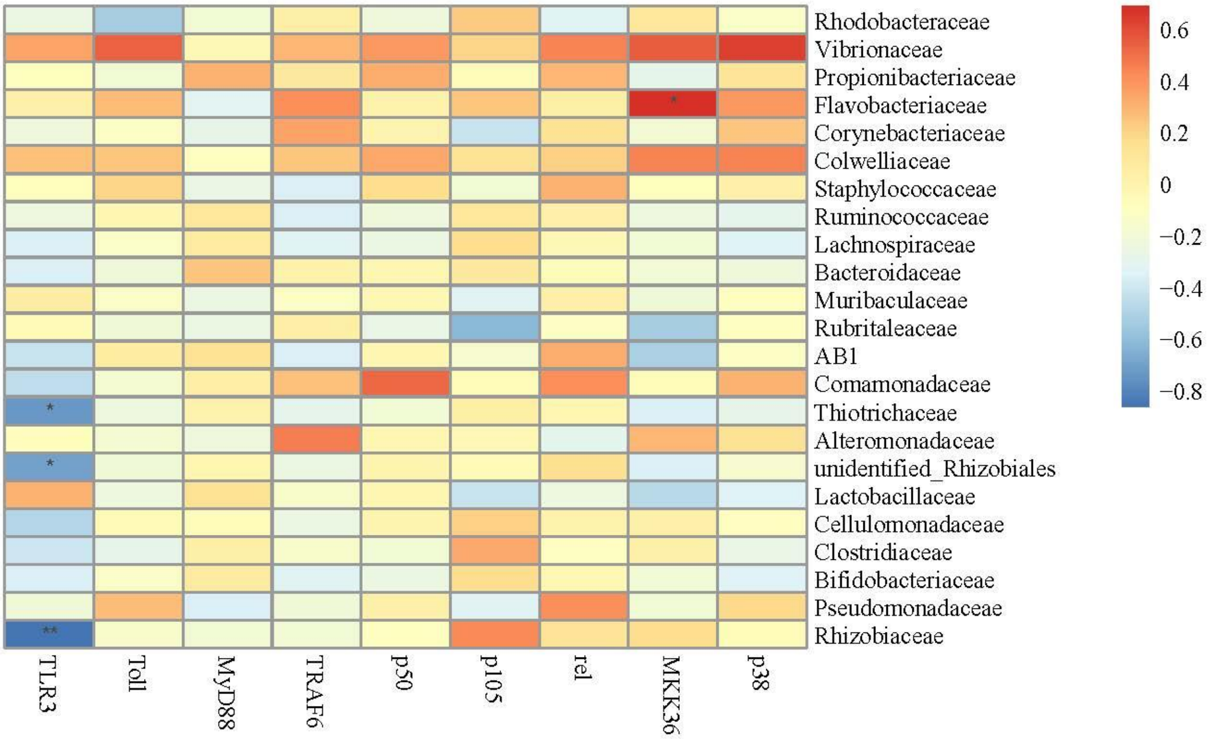

Figure S3 Correlations between the immune related genes and top 20 families and major biomarker families of microbial community in intestine of the *A. japonicus*. (* $P < 0.05$, ** $P < 0.01$).
